# Structural and Functional Insights Into CmGH1, a Novel GH39 Family β-Glucosidase From Deep-Sea Bacterium

**DOI:** 10.3389/fmicb.2019.02922

**Published:** 2019-12-20

**Authors:** Yanfang Shen, Zhengyang Li, Ying-Yi Huo, Luyao Bao, Baocai Gao, Peng Xiao, Xiaojian Hu, Xue-Wei Xu, Jixi Li

**Affiliations:** ^1^State Key Laboratory of Genetic Engineering, Department of Neurology, School of Life Sciences, Huashan Hospital, Shanghai Engineering Research Center of Industrial Microorganisms, Fudan University, Shanghai, China; ^2^Key Laboratory of Marine Ecosystem and Biogeochemistry, Second Institute of Oceanography, State Oceanic Administration, Hangzhou, China

**Keywords:** CmGH1, β-glucosidase, crystal structure, characterization, *Croceicoccus marinus*, marine bacterium

## Abstract

Glucosidases play key roles in many diseases and are limiting enzymes during cellulose degradation, which is an important part of global carbon cycle. Here, we identified a novel β-glucosidase, CmGH1, isolated from marine bacterium *Croceicoccus marinus* E4A9^T^. In spite of its high sequence and structural similarity with β-xylosidase family members, CmGH1 had enzymatic activity toward *p*-nitrophenyl-β-D-glucopyranoside (*p-*NPG) and cellobiose. The *K*_m_ and *K*_cat_ values of CmGH1 toward *p-*NPG were 0.332 ± 0.038 mM and 2.15 ± 0.081 min^–1^, respectively. CmGH1 was tolerant to high concentration salts, detergents, as well as many kinds of organic solvents. The crystal structure of CmGH1 was resolved with a 1.8 Å resolution, which showed that CmGH1 was composed of a canonical (α/β)_8_-barrel catalytic domain and an auxiliary β-sandwich domain. Although no canonical catalytic triad residues were found in CmGH1, structural comparison and mutagenesis analysis suggested that residues Gln157 and Tyr264 of CmGH1 were the active sites. Mutant Q157E significantly increased its hydrolase activity up to 15-fold, whereas Y264E totally abolished its enzymatic activity. These results might provide new insights into understanding the different catalytic mechanism during evolution for β-glucosidases and β-xylosidases.

## Introduction

β-xylosidases (or xylan 1,4-β-xylosidases, EC 3.2.1.37) catalyze the hydrolysis of short chain xylooligosaccharide into xylose units, which play an important role in plant cell-wall hemicelluloses degradation and carbon cycle maintenance (Beg et al., 2001; Galbe and Zacchi, 2002). The depolymerization xylan to free xylose has a significant relevance for lignocellulose-based biofuels production by pentose-fermenting microorganisms and other industrial processes such as the paper and food industries (Bosetto et al., 2016). According to the amino acid sequence and structure, β-xylosidases are generally assigned to glycoside hydrolase (GH) families 1, 3, 30, 39, 43, 51, 52, 54, 116, and 120 (Henrissat and Davies, 1997). With the development of industry, it is of great interest to explore different β-xylosidases with compatible stability in harsh conditions from bacteria and fungi (Joseleau et al., 1992; Jordan and Wagschal, 2010; Lagaert et al., 2011).

*Croceicoccus marinus* E4A9^T^, the type species of the genus *Croceicoccus*, was isolated from deep-sea sediment at the East Pacific polymetallic nodule region (5,280 m depth, 2°C, 34 ‰ salinity) (Xu et al., 2009). The enzymes from marine bacteria might have good stress tolerance (Jiang et al., 2012; De Santi et al., 2016; Huang et al., 2016). A novel glycosidase gene *cmgh1* was identified based on the *in silico* analysis (Kim et al., 2004; de Pascale et al., 2008). Sequence analysis of the *cmgh1* gene showed that it shared 51% identity with the β-xylosidase from *Microbacterium testaceum* StLB037^T^, indicating that CmGH1 might be a new β-xylosidase belonging to the GH39 family (Morohoshi et al., 2011).

Different with β-xylosidase, the β-glucosidases (or β-D-glucoside glucohydrolase, EC 3.2.1.21) have specific substrate-binding pattern and catalytic activities, and facilitate release of aromatic compounds, phytohormone activation, as well as cell wall oligosaccharide recycling that hydrolyze exogenous glucosides, glycolipids and oligosaccharides into β-D-glucose in microbials, animals and plants (Bhatia et al., 2002; Ketudat Cairns and Esen, 2010; Ketudat Cairns et al., 2015). β-glucosidases play key roles in the synthesis or hydrolysis of glycosidic bonds, which is the rate-limiting step in sugar metabolism and biofuels production (Bhatia et al., 2002; Singhania et al., 2013; Kuusk and Valjamae, 2017). β-glucosidases are well-characterized and generally classified into many GH families 1, 2, 3, 5, 9, 30, 39, and 116 (Henrissat and Davies, 1997). However, there is no β-glucosidase reported in GH39 family and the structural basis for substrate specificity is barely investigated. Here, we present the characterizations and crystal structure of CmGH1, which showed β-glucosidase activity, instead of β-xylosidase activity, to provide a structural basis for the development, and utilization of the marine sourced β-glucosidase.

## Results

### Biochemical Properties of CmGH1

**FIGURE 1:**
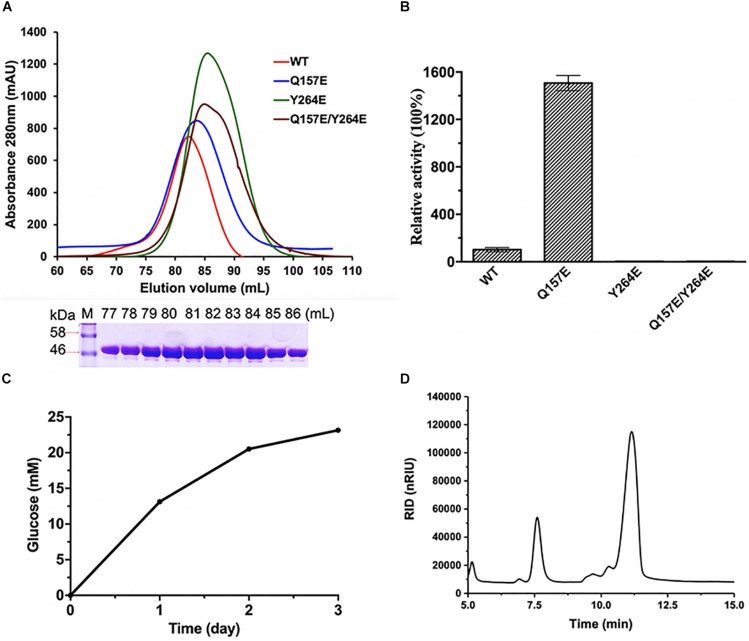
Purification and enzymatic activities of CmGH1 and its mutants. **(A)** (Top) Gel filtration chromatography of CmGH1 and its mutants on Superdex 200 16/600 columns. The CmGH1 protein was eluted at the peak of 82 mL (bottom) SDS-PAGE of CmGH1 protein eluted from gel filtration. The left lane was the molecular weight marker (labeled in kDa). **(B)** The enzymatic activities of CmGH1 and its mutants toward *p*-nitrophenyl-β-D-glucopyranoside. The value of CmGH1-WT was taken as 100%. **(C,D)** The enzymatic activity of CmGH1 toward cellobiose was analyzed by HPLC method. The concentration of glucose (product) was detected. **(D)** Cellobiose (substrate) and glucose (product) came out at the peak positions of 11.165 min and 7.592 min on a ZORBAX NH_2_ column, respectively.

A putative open reading frame of 1458 bp (*cmgh1*), encoding a protein of 485 aa (CmGH1) with a theoretical molecular weight of 53.48 kDa and pI of 4.66, was identified from the whole-genome of strain *C. marinus* E4A9^T^. According to the phylogenetic tree, the protein CmGH1 belongs to the GH39 family (Supplementary Figure S1). To investigate the catalytic characterization of CmGH1, the recombinant protein CmGH1 with a N-terminal His_6_-SUMO tag was expressed in *Escherichia coli* BL21 (DE3) cells. After removal of the His_6_-SUMO tag with Ulp1 enzyme as described in previous study (Kuang et al., 2017), the target protein CmGH1 was purified into 95% homogeneity by gel filtration chromatography (Figure 1A). CmGH1 was firstly predicted to be a β-xylosidase based on sequence analysis, however, CmGH1 did not show β-xylosidase activity when using *p*-nitrophenyl-β-D-xylopyranoside, xylobiose and xylotriose as substrates, with the high sensitive HPLC method and 3,5-dinitrosalicylic acid (DNS) method (McCleary and McGeough, 2015). Therefore, CmGH1 was evaluated for saccharification potential with different substrates, which turned out that CmGH1 showed β-glucosidase activity when using *p*-nitrophenyl-β-D-glucopyranoside (*p-*NPG) and cellobiose as substrates (Figures 1B–D and Supplementary Figure S2).

**FIGURE 2:**
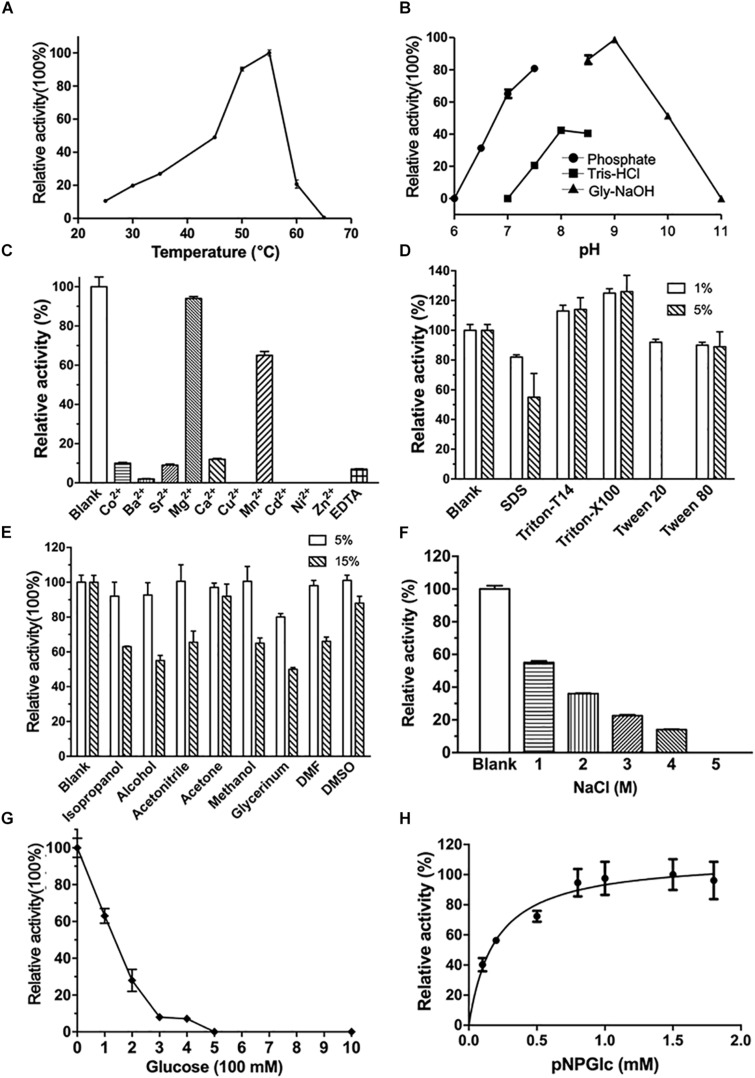
Enzymatic characterization of CmGH1. **(A)** Effects of temperature on enzyme activity. The value obtained at 55°C was taken as 100%. **(B)** Effects of pH on enzyme activity. The value obtained at pH 9.0 was taken as 100%. **(C)** Effects of different metal ions on the enzymatic activity. The values obtained without ions in the reaction mixture were taken as 100%. **(D)** Effects of different detergents on the enzymatic activity. All kind of detergents were added at the final concentration of 1% and 5%, respectively. The values obtained without ions in the reaction mixture were taken as 100%. **(E)** Effects of different organic solvents on the enzymatic activity. All kind of organic solvents were added at the final concentration of 5% and 15%, respectively. The values obtained without ions in the reaction mixture were taken as 100%. **(F)** Effects of NaCl concentration on the activities. The value obtained without NaCl in the reaction mixture was taken as 100%. **(G)** Effects of products (glucose) concentration on the activities. The value obtained without glucose in the reaction mixture was taken as 100%. **(H)** Effects of substrate *p*-nitrophenyl-β-D-glucopyranoside on the activities. The value obtained with 1.5 mM *p*-nitrophenyl-β-D-glucopyranoside was taken as 100%.

The optimum reaction conditions of CmGH1 toward *p-*NPG were determined over a pH range of 6.0–11.0 and a temperature range of 25–65°C. CmGH1 showed the highest catalytic activity at pH 9.0 and 55°C (Figures 2A,B). The enzyme activity of CmGH1 was 1.46 × 10^–2^ U/mg, and the *K*_m_ and *K*_cat_ values were 0.332 ± 0.038 mM and 2.15 ± 0.081 min^–1^, respectively.

Furthermore, various kinds of divalent cations, organic solvents and detergents were added into the reaction buffer to investigate the tolerance of CmGH1 (Figures 2C–F). The β-glucosidase activity of CmGH1 was completely abolished with the additions of Zn^2+^, Ni^2+^, Cd^2+^, Cu^2+^, and Ba^2+^, and only about 10–15% activity was remained with Ca^2+^, Sr^2+^, and Co^2+^. CmGH1 could tolerate Mn^2+^ and Mg^2+^ with the relative activity values at 64% and 88%, respectively. In addition, 10 mM EDTA severely suppressed the activity of CmGH1 which remained 10% relative activity (Figure 2C). Most detergents and organic solvents had little impacts on the catalytic reaction of CmGH1. The β-glucosidase activities were comparative with the blank under the addition of 1% detergents (Triton X-T14, Triton X-100, Tween20, Tween80, and SDS) and 5% organic solvents (DMSO, DMF, glycerinum, methanol, acetone, acetonitrile, alcohol, and isopropanol). The activities attenuated significantly under conditions with SDS or Tween20 at 5% concentration, as well as glycerinum, alcohol, isopropanol, DMF, methanol, and acetonitrile at 15% concentrations. However, 15% DMSO and acetone displayed little effects on the activity of CmGH1 (Figures 2D,E). Moreover, CmGH1 had enzymatic activity (about 30%) with addition of 2 M NaCl, but abolished its activity at higher salt concentration (Figure 2F). CmGH1 remained over 30% enzymatic activity with the presence of 200 mM glucose, however, completely abolished its activity at 500 mM or higher concentration (Figure 2G). In addition, CmGH1 reached its maximum activity with 1.0 mM *p*-NPG or higher concentration (Figure 2H).

### Overall Structure of CmGH1

The crystal structure of CmGH1 was solved with a 1.8 Å resolution (Table 1). Diffraction dataset was integrated into the monoclinic space group P2_1_ with two molecules per asymmetric unit. The two chains were identical and the root-mean-square deviation (RMSD) value for its backbone was 0.211 Å. The structure of CmGH1 was refined to the satisfied *R*_work_ and *R*_free_ values of 14.71% and 18.94%, respectively. The first four residues at the N-terminal region and the residues from Val396 to Thr403 were invisible in chain A but were refined unambiguously in the electron-density map of chain B. Therefore, we will discuss the Chain B structure only.

**TABLE 1:** Data collection and refinement statistics of CmGH1.

**Items**	**CmGH1-Se**
**Data collection**	
Wavelength	0.9793
Resolutions (Å)	48.35–1.80 (1.85–1.80)^a^
Space group	P 2_1_
Unit cell (Å,°)	*a* = 48.911, *b* = 95.911, *c* = 100.908 α = γ = 90, β = 98.712
Unique reflections	82639 (8048)
Completeness (%)	97.31 (95.41)
*R*_merge_ (%)^b^	13.7 (58.5)
I/σ (I)	15.03 (3.67)
Number of Se atoms	18
**Refinement statistics**	
Resolutions (Å)	48.35–1.80 (1.85–1.80)
Reflection used in refinement	82639 (8048)
*R*_work_ (%)^c^	14.71 (26.14)
*R*_free_ (%)^d^	18.94 (31.82)
Number of glycerol	2
Number of water	560
Number of protein residues	961
**RMSD**	
Bond lengths (Å)	0.007
Bond angles (°)	0.84
Average B-facor (Å^2^)	19.75
Ramachandran favored (%)	96.43
Ramachandran allowed (%)	3.57
Ramachandran outliers (%)	0
Rotamer outliers (%)	0
PDB code	5Z3K

*^*a*^Statistics for the highest-resolution shell are shown in parentheses. ^*b*^*R*_*merge*_ = Σ | Ii - ı| /Σ | I|, where I_*i*_ is the intensity of an individual reflection and I is the average intensity of that reflection. ^*c*^*R*_*work*_ = Σ | | F_*o*_| - | F_*c*_| | /Σ | F_*o*_|, where F_*o*_ and F_*c*_ are the observed and calculated structure factors for reflections, respectively. ^*d*^*R*_*free*_ was calculated as *R*_*work*_ using the 5% of reflections that were selected randomly and omitted from refinement.*

**FIGURE 3:**
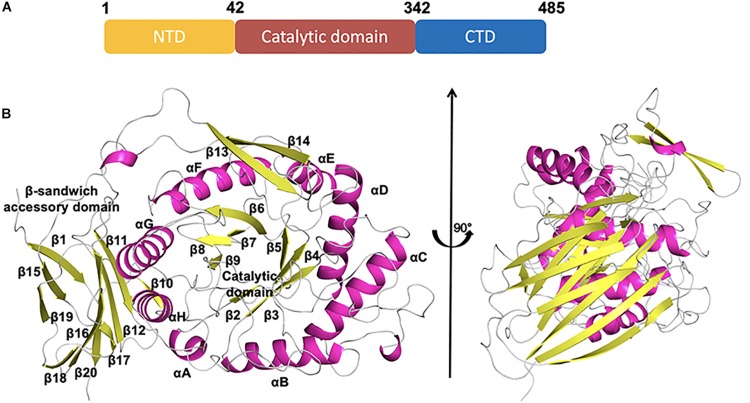
Schematic representation of CmGH1 structure. **(A)** Schematic of CmGH1 protein, which was composed of the N-terminal domain (NTD), the catalytic domain, and the C-terminal domain (CTD). **(B)** Cartoon representation of CmGH1 with the labeled secondary structure elements. β-strands were denoted as yellow arrows; α-helices were shown in magenta.

CmGH1 was composed of the N-terminal domain (NTD), the C-terminal domain (CTD), and the middle catalytic domain (Figure 3A). The CmGH1 structure had eight α-helices and twenty β-stands, which combined into a classical (α/β)_8_-barrel catalytic core domain and a β-sandwich accessory domain (Figure 3B). The core domain included eight parallel β-stands, namely β2 (Leu53-Arg55), β3 (Asn104-Ile109), β4 (His151-Phe154), β5 (Lys191-Val197), β6 (Phe223-Tyr229), β7 (Glu259-Leu262), β7 (Tyr264-Ser266), and β9 (Lys298-Phe301), surrounded by eight α-helices, namely αA (Leu43-Asn48), αB (Tyr86-Ser100), αC (Leu123-Val138), αD (Pro169-Val186), αE (Glu208-Gln216), αF (Asp238-Lys252), αG (Asp277-Met291), and αH (Ala329-Leu341). A β-hairpin motif consisting of β13 (Val391-Ile398), β14 (Gln401-Asn408) and the loop between these two β-stands protruded out from the barrel. The accessory domain included β1 (Ile6-Asn12), β10 (Thr344-Arg346), β11 (Phe357-Arg363), β12 (Thr369-Asn376), β15 (Gly423-Gly429), β16 (His438-Asp446), β17 (His449-Glu452), β18 (Leu454-Gly459), β19 (Val464-Lys469), and β20 (Gly474-Arg482) (Figure 3B).

### Structural Comparison of CmGH1 With Other GH39 Family Members

**FIGURE 4:**
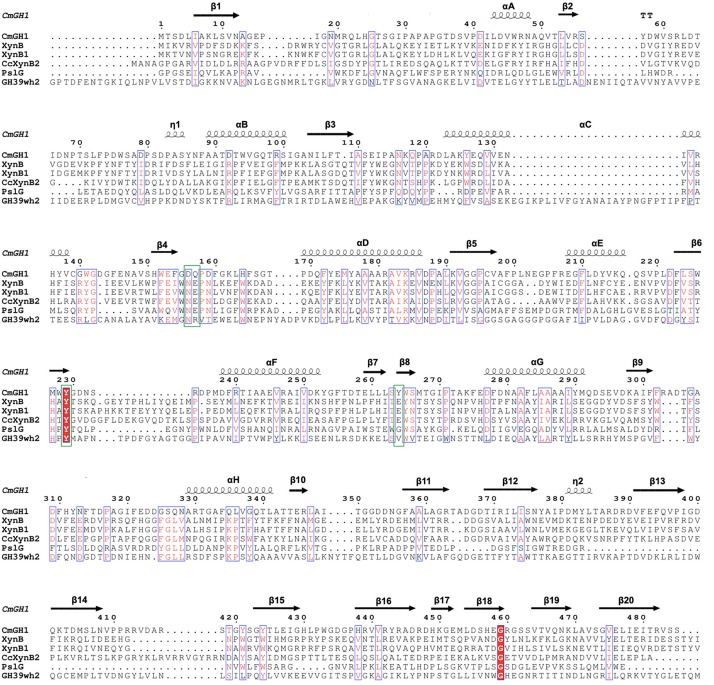
Multiple sequence alignment of CmGH1 with other GH39 family β-xylosidases. The sequences include XynB from *T. saccharolyticum* [PDB_ID: 1PX8 (Yang et al., 2004)], XynB1 from *G. stearothermophilus* (PDB_ID: 1W91), CcXynB2 from *Caulobacter crescentus* [PDB_ID: 4EKJ (Santos et al., 2012)], PslG from *P. aeruginosa* [PDB_ID: 4ZN2 (Yu et al., 2015)], and GH39wh2 from *Bacteroides cellulosilyticus* [PDB_ID: 5JVK (Ali-Ahmad et al., 2017)]. Residues that might contribute to catalytic center were labeled by green box.

**FIGURE 5:**
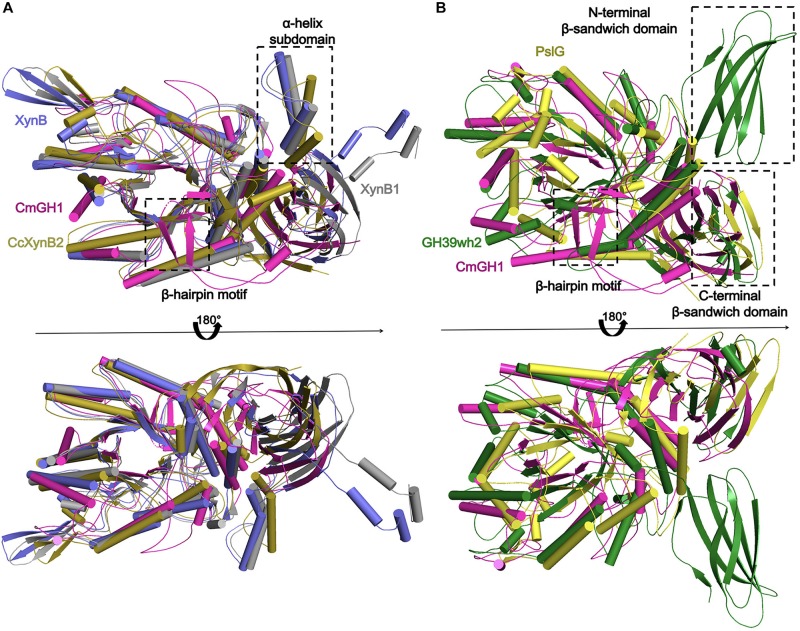
Structural comparison of CmGH1 with other homologs of GH39 family. **(A)** The structural superposition of CmGH1 (magenta), XynB (blue, PDB_ID: 1PX8 (Yang et al., 2004), RMSD: 5.171 Å, 206 atoms), XynB1 (gray, PDB_ID: 1W91, RMSD: 1.925 Å, 147 atoms), and CcXynB2 [dark yellow, PDB_ID: 4EKJ (Santos et al., 2012), RMSD: 9.550 Å, 236 atoms]. **(B)** The structural superposition of CmGH1 (magenta), GH39wh2 [green, PDB_ID: 5JVK (Ali-Ahmad et al., 2017), RMSD: 10.275, 263 atoms], and PslG [yellow, PDB_ID: 4ZN2 (Yu et al., 2015), RMSD: 11.294, 240 atoms].

Several GH39 family β-xylosidase crystal structures have been reported, including XynB from *Thermoanaerobacterium saccharolyticum* [PDB_ID: 1PX8 (Yang et al., 2004)], XynB1 from *Geobacillus stearothermophilus* (PDB_ID: 1W91), CcXynB2 from *Caulobacter crescentus* [PDB_ID: 4EKJ (Santos et al., 2012)], PslG from *Pseudomonas aeruginosa* [PDB_ID: 4ZN2 (Yu et al., 2015)] and GH39wh2 from *Bacteroides cellulosilyticus* (PDB_ID: 5JVK [Ali-Ahmad et al., 2017)]. These structures share similar architectures, including a conservative (α/β)_8_-barrel catalytic domain and a β-sandwich domain at the C-terminal region (Figures 4, 5A,B). Moreover, XynB and XynB1 have an additional long α-helix-containing auxiliary domain at the C-terminal region (Yang et al., 2004; Czjzek et al., 2005; Santos et al., 2012).

Structural superimposition showed that CmGH1 shared a similar architecture with other GH39 family β-xylosidases, despite that PslG and GH39wh2 only shared 17% and 13% identities with CmGH1 (Figures 4, 5A,B). PslG has one conservative (α/β)_8_-barrel catalytic domain and one β-sandwich domain at C-terminal region, whereas GH39wh2 has one (α/β)_8_-TIM barrel and two β-sandwich domains at N-terminal and C-terminal region. The N-terminal β-sandwich domain could afford minimal flexibility requiring to provide an additional substrate-binding interface and likely to strengthen substrate recognition (Ali-Ahmad et al., 2017; Figure 5B). In addition, structural comparisons among CmGH1, RlGH from *Rhizobium leguminosarum* (PDB_ID: 5NDX), and CvGH from *Caulobacter vibrioides* (PDB_ID: 4M29) showed that CvGH shared similar architecture with CmGH1, whereas RlGH had one more unknown function β-sandwich domain at C-terminal region (Supplementary Figure S3).

### Catalytic Center and Active Sites of CmGH1

**FIGURE 6:**
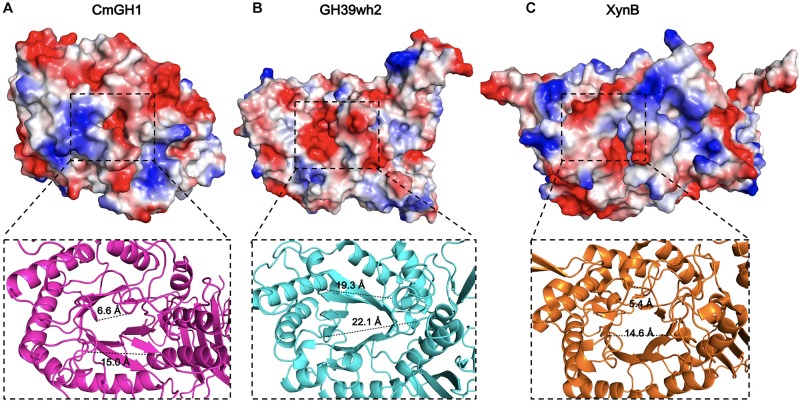
The active site regions of CmGH1 and its homologs. The electrostatic potential surfaces of CmGH1, GH39wh2 [PDB_ID: 5JVK (Ali-Ahmad et al., 2017)] and XynB [PDB_ID: 1PX8 (Yang et al., 2004)] were shown in **(A–C)** (top). Red, negative potential; Blue, positive potential. The zoomed grooves were shown in the bottom. The distance was measured between the Cα atom of the nearest residues of the facing loops across the groove, which was shown as dashed lines.

The catalytic pocket of GH39 family enzymes, such as PslG and GH39wh2, are surrounded by negative charged residues (Ali-Ahmad et al., 2017). The corresponding area in CmGH1 showed the same predominant negative surface potential cluster (Figure 6). Among GH39 family members, PslG and GH39wh2 acted as endoglycosidase with a large groove, whereas XynB, XynB1, CcXynB2, and α-L-iduronidase might act as exoglycosidase because of the small groove, which can only accept oligosaccharides as substrates (Yang et al., 2004; Santos et al., 2012). The groove distances among residues Phe114 and Tyr312, Pro271 and Leu163 of CmGH1 were 6.6 Å and 15.0 Å, respectively, whereas the corresponding distances in the groove of GH39wh2 were 19.3 Å and 22.4 Å (Tyr210 and Glu415, Asn251, and Asn370), and those in XynB were 5.4 Å and 14.6 Å (Tyr116 and Arg324, Tyr282 and Phe166) (Figure 6). Therefore, CmGH1 might be a new exoglycosidase when comparing with GH39wh2 and XynB.

**FIGURE 7:**
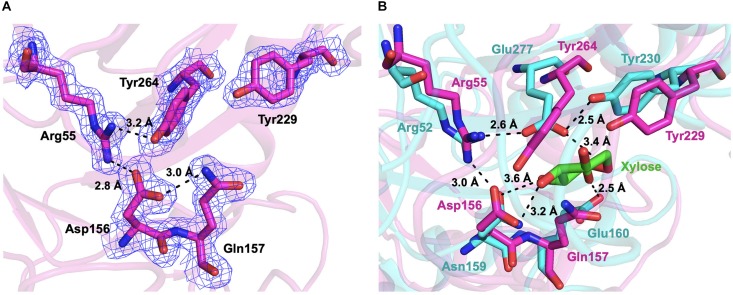
The active sites of CmGH1. **(A)** The active sites were shown in the stick model. The interactions and distances within these residues were labeled in black dashed lines. The electrostatic map was contoured to 1.0 σ at the 2*Fo-Fc* map. **(B)** Superposition of the active sites of CmGH1 (magenta) and XynB [cyan, PDB_ID: 1PX8 (Yang et al., 2004)]. The substrate of XynB, D-xylose, was shown in green sticks.

The active sites of CmGH1 might locate within the central (α/β)_8_ TIM barrel by structure comparison with other members. The active sites of XynB include the conserved Glu160 and nucleophile Glu277, whereas the corresponding residues in CmGH1 were Gln157 and Tyr264 (Figures 4, 7). Although no glutamic acid was found in the catalytic pocket of CmGH1, it might use a similar catalytic mechanism with the other members of GH39 family. Besides the active sites, CmGH1 did not have any conserved substrate binding residues with those reported in XynB (Ile151-Leu164) (Figure 4). When docking and computing CmGH1 with different substrates (cellobiose and xylobiopyranose) and products (D-glucose and D-xylopyranose) using AutoDock Tools4 program (Morris et al., 2009), the binding energy and inhibition constants of the CmGH1 complexes with cellobiose and glucose were lower than that with xylobiopyranose and xylopyranose, which meant CmGH1 might prefer to use cellobiose and glucose as substrates (Supplementary Figure S4 and Supplementary Table S1).

To verify the possible active sites of CmGH1, site-directed mutagenesis was performed to identify their roles in enzymatic activity. The mutant Q157E had 15 folds higher catalytic activity toward *p*-NPG than wild-type CmGH1, whereas the mutant Q157A had a similar value with wild-type CmGH1. The enzyme activity of CmGH1-Q157E was 2.04 × 10^–1^ U/mg, while the *Km*, Vmax, and *K*_cat_ values were 0.3542 ± 0.042 mM, 109.5 ± 3.29 μM/min, and 29.28 ± 0.88 min^–1^, respectively. However, Y264E and Q157E/Y264E mutants abolished the enzymatic activities of CmGH1, which suggested that Tyr264 could contribute a vital role in the enzyme activity (Figure 1B).

## Discussion

Glycosidases are a group of evolutional conserved enzymes that cleave the glycosidic bonds and have been classified into over 63 families according to their sequences (Sinnott, 1990; Henrissat and Bairoch, 1996). Based on substrate specificity, glycosidases are consisted of β-xylosidase, β-D-glucosidase, β-mannosidase, myrosinase, and so on (Mian, 1998). Although CmGH1 was predicted as a β-xylosidase, the biochemical results suggested CmGH1 might be a β-D-glucosidase belonging to the GH39 family. The auto-docking result also showed that CmGH1 preferred to combine cellobiose and glucose, which had lower binding energy and inhibition constant compared with xylobiopyranose and xylopyranose. Most characterized β-D-glucosidase showed highest activity in the acidic pH range and remained low activity under alkaline environment (Bhatia et al., 2002; Sorensen et al., 2013). CmGH1 had maximal activity at pH 9.0 and remained over 50% activity at pH 10.0 (Figure 2B). This uncommon feature might be due to the host line *C. marinus*, which lives in the marine environment and prefers a mildly alkaline condition. This property might have a potential application in many industrial processes of which the high pH catalytic condition required. The catalytic activity of Q157E was 15 times higher than that of wild type CmGH1, most probably because that Gln157 acted as an auxiliary catalytic proton donor and glutamine had a weaker proton donating ability than glutamic acid (Brooks et al., 2001; Vocadlo et al., 2002). The bacteria *C. marinus* E4A9^T^ might prefer a weaker acid/base residue to keep an insufficient catalytic activity, which was related to the evolution divergence and was an adaptive selection for the extreme environment in deep sea (Xu et al., 2009).

As Y264E and Q157E/Y264E mutants abolished the enzymatic activities (Figures 1A,B), this suggested that Tyr264 might play a vital role in the catalytic site. In the GH83 and GH143 families, one Tyr residue was reported as nucleophile in the catalytic center. In corresponding with Glu277 of XynB, the Tyr264 of CmGH1 could act as a nucleophile during catalysis (Crennell et al., 2000; Ndeh et al., 2017).

As CmGH1 had the optimum pH at 9.0 (Gly-NaOH buffer) and abolished the enzymatic activities at acidic conditions (pH < 6.0), we suggest that Tyr264 might act as a nucleophile residue only under alkaline conditions. Although it is not clear how many amino acids are involved in the catalytic process of CmGH1, we found that Asp156, Glu157, Tyr229, Arg55, and Tyr264 might play a major role in catalytic reaction by superimposition with the substrate D-xylose in XynB (Figures 4, 7).

The superimposed CmGH1 with other members of GH39 families showed that there were some differences in architectures, apart from the conserved (α/β)_8_-TIM barrel domain and β-sandwich domain. For example, the β-hairpin motif of CmGH1 extended from the catalytically active pocket and held onto one end, which was not reported in GH39 family. In CcXynB2, there was a β-hairpin motif connecting the sixth loop β7 to αF and interacted with a long α-helix-containing loop that was the only one of the reported xylosidases of GH39 through polar and hydrophobic effects, which induced the β-hairpin motif adopting an open conformation (Santos et al., 2012). However, the β-hairpin motif of CmGH1 was far away from the nearby small α-helix, indicating that there was no interaction between them and the β-hairpin motif adopted a closed conformation, which might be related to its substrate recognition (Figure 5).

The substrate-binding groove in CmGH1 was slightly larger than that in XynB and smaller than that in GH39wh2, which could be inferred by different states, as XynB and XynB1 appeared to be a tetramer in solution, whereas CcXynB2, PsIG, and GH39wh2 formed monomers in solution (Yang et al., 2004; Czjzek et al., 2005; Santos et al., 2012; Baker et al., 2015; Ali-Ahmad et al., 2017). In addition, the GH1 family members share (α/β)_8_ barrel catalytic domain and use Glu as the catalytic residue, whereas the GH3 family members have no specific catalytic domain and use Glu or His or Asp as catalytic acid-base and nucleophile residue, respectively (Rye and Withers, 2000; Vasella et al., 2002). By compared with these glucosidases, CmGH1 did not belong to any known family of β-D-glucosidases.

In conclusion, CmGH1 showed glucosidase activity toward different substrates *p*-NPG and celloboise, despite of that it shared sequence similarity with xylosidase. These findings provide new insights into understanding the different catalytic mechanism during evolution for β-glucosidases and β-xylosidases, as well as offer a structural and theoretical basis for modification of industrial enzymes.

## Materials and Methods

### Bacterial Strains, Plasmids, and Media

*Croceicoccus marinus* E4A9^T^, a member of *Erythrobacteraceae* family, was isolated from a deep-sea sediment sample that was collected from the East pacific polymetallic nodule region, and was cultivated in 2216 marine broth (BD, United States) at 30°C (Xu et al., 2009). Plasmid pSMT3 was stored in our lab and used as the vector for gene cloning and expression. *E. coli* strain DH5α and BL21 (DE3) plysS (Transgen, China) were used as the host for cloning and protein expressing. *E. coli* cells were grown at 37°C in LB medium containing 10 g NaCl, 10 g tryptone and 5 g yeast extract (Sigma-Aldrich, United States) per liter at pH 7.0, and LB agar medium was added with 1.5–2.0% (w/v) agar.

### Sequence Analysis

Multiple alignment of amino acid sequences of homologs was performed using ClustalX v.2 program (Larkin et al., 2007). Second structure alignment was generated by the ESpript v.3.0 server (Robert and Gouet, 2014). The phylogenetic tree was constructed by neighbor-joining method using MEGA (Molecular Evolutionary Genetics Analysis) v.10.1 software (Felsenstein, 1985; Saitou and Nei, 1987; Kumar et al., 2018).

### Cloning, Protein Expression, and Purification

The gene *cmgh1* (accession number: WP_066849948) from *C. marinus* was inserted into the pSMT3 vector. The wild type and mutants of CmGH1 plasmids were transformed into *E. coli* BL21 (DE3) plysS cells for protein expression. 0.5 mM isopropyl β-D-thiogalactoside was added into cells to induce protein expression at 16°C for 20 h when the OD_600_ reached 0.8. Then the cells were harvested by centrifugation at 6000 rpm for 10 min at 4°C. The cell pellets were resuspended in a lysis buffer (50 mM Tris–HCl, 500 mM NaCl, 10 mM imidazole, 5% glycerol, 2 mM β-ME, pH 8.0) and were disrupted using a high-pressure homogenizer. The supernatant was purified by NTA affinity chromatography. After cleavage of the His_6_-sumo tag with Ulp1 enzyme, the target proteins were obtained in the flow-through fractions. Subsequently, CmGH1 protein was purified by gel-filtration chromatography (Superdex 200 16/600, GE, United States). The fractions were determined by SDS-PAGE and the concentration was measured by the method of Bradford with bovine serum albumin (BSA) as a standard (Bradford, 1976).

Selenomethionine (Se-Met) substituted CmGH1 protein was expressed as mentioned above. When OD_600_ reached 0.4, the cells were harvested by centrifugation and resuspended in 100 mL M9 medium (47.7 mM Na_2_HPO_4_, 22 mM KH_2_PO_4_, 8.6 mM NaCl, and 28.2 mM NH_4_Cl). The resuspended cells were centrifuged and transferred into 500 mL fresh M9 medium supplemented with 50 μg/mL kanamycin and 30 mg/L Se-Met. After growing at 37°C for 1 h, the temperature was lowered to 16°C and the protein expression was induced by adding 0.5 mM IPTG for an additional 18 h. Then, the cells were harvested by centrifugation and purified as mentioned above.

### Biochemical Characterization of CmGH1

The enzymatic activity of wild type CmGH1 and mutants were tested by *p*-nitrophenol method and DNS method (McCleary and McGeough, 2015). The enzymatic activities toward xylopyranoside and glucopyranoside were measured using *p*-nitrophenyl-β-D-xylopyranoside (*p-*NPX) and *p*-nitrophenyl-β-D-glucopyranoside (*p-*NPG) as substrates, respectively. The standard reaction buffer consisted of 100 μM purified CmGH1 and 1mM *p-*NPX (or *p-*NPG) in 200 ul buffer with different pH varying from 3.0 to 11.0, including 100 mM citrate buffer (pH 3.0-pH 6.5), 100 mM potassium phosphate buffer (pH 6.5-pH 7.5), 100 mM Tris–HCl buffer (pH 7.5-pH 9.0), and 100 mM Gly-NaOH buffer (pH 9.0-pH 11.0). The enzyme activity was determined by measuring the amount of released *p*-nitrophenol from 293K to 333K at 405 nm using SpectraMax M5 (Molecular Devices, United States). The absorbance of 405 nm was measured every 5 min and the total reaction time is 5 h.

The influences of cations on enzyme activity were examined in the presence of 10 mM Ba^2+^, Ca^2+^, Co^2+^, Cu^2+^, Mg^2+^, Mn^2+^, Ni^2+^, Sr^2+^, Zn^2+^, and the chelating agent EDTA. The effects of organic solvents were tested by using 5% and 15% isopropanol, alcohol, acetonitrile, acetone, methanol, glycerinum, dimethylformamide (DMF), or dimethyl sulfoxide (DMSO). The effects of detergents were determined by using 1% or 5% Tween20, Tween80, Triton X-100, or SDS. All measurements were performed in 100 mM Tris–HCl buffer (pH 7.5), and the enzyme activity in the blank group was defined as 100% without additives. The salt tolerance of CmGH1 was determined by adding 1 M, 2 M, 3 M, 4 M or 5 M NaCl to 100 mM Gly-NaOH buffer (pH 9.0). The enzyme activity in the blank group was defined as 100% without NaCl. The tolerance of products on enzymatic activities was measured in the presence of 0.1, 0.2, 0.3, 0.4, 0.5, and 1 M glucose. The influences of substrate were determined by adding 0.1, 0.2, 0.5, 0.8, 1.0, 1.5, and 1.8 mM *p-*NPG.

In the reaction system of DNS method, the amount of xylobiose or xylotriose was 1 mM, and the amount of CmGH1 was 0.1, 1, 10, and 50 mM, respectively. The reaction buffer pH varied from 3.0 to 11.0, including 100 mM citrate buffer (pH 3.0 -pH 6.5), 100 mM potassium phosphate buffer (pH 6.5 -pH 7.5), 100 mM Tris–HCl buffer (pH 7.5-pH 9.0), and 100 mM Gly-NaOH buffer (pH 9.0 -pH 11.0). The reaction temperature was 20–60°C, and the reaction time was 10 min, 30 min, 1 h, 2 h, 12 h, 24 h, 48 h, and 1 week. DNS reagents, which contained 10 g 3,5-dinitrosalicylic acid, 2 g phenol, 0.5 g sodium sulfide, 10 g sodium hydroxide and 80 g potassium sodium tartrate, were added to the completed reaction system and incubated in boiling water bath for 5 min. Then the solution was quickly cooled to room temperature. The reaction mixture was measured with the absorbance at 540 nm wavelength using SpectraMax M5. The standard curve was measured with xylose at various concentration according to the above experimental method and the linear equation was Y = 0.6567X + 0.0643, *R*^2^ = 0.9984.

The HPLC method was used to detect enzymatic activity of CmGH1 toward cellobiose and xylobiose. The reaction mixture contained 250 mM cellobiose (or 170 mM xylobiose), 1 mg/mL CmGH1 and 100 mM Gly-NaOH buffer (pH 9.0). The catalytic reaction was performed at 55°C for various times (1, 2 and 3 days for cellobiose, and 12 h for xylobiose). BSA (1mg/mL), instead of CmGH1, was added into the reaction mixture as a negative control. Ethanol was added into the mixture to quench this reaction. The samples were centrifuged for 10 min at 17,000 g and the supernatants were detected by HPLC on a ZORBAX Original 70 Å NH_2_ column (Agilent, United States) with 75% acetonitrile as a mobile phase. The concentration of glucose was calculated by using the standard curve, Y = 3 × 10^–5^X + 1.1617 (Y: glucose concentration, X, peak area, *R*^2^ = 0.9978).

### Crystallization and Data Collection

The Se-Met substituted CmGH1 protein was crystallized using the hanging drop vapor diffusion method at 291K by mixing 1 μL of 5 mg/mL protein with 1 μL of reservoir solution, including 0.2 M ammonium acetate, 0.1 M Bis-Tris pH 5.5 and 25% PEG3350. Diffraction data were collected with crystals flashing-frozen in crystallization buffer supplemented with 25% (v/v) glycerol. The Se-Met derivative data sets were collected at BL17U1, 18U1, and 19U1 beamlines of the Shanghai Synchrotron Radiation Facility (Wang et al., 2018). Diffraction data were integrated and scaled using software HKL2000 or HKL3000 (Otwinowski and Minor, 1997; Minor et al., 2006).

### Structure Determination and Refinement

The structure of CmGH1 was solved by single wavelength anomalous diffraction (SAD) method with a resolution 1.8 Å. In brief, the diffraction data were scaled with XDS and merged using AIMLESS from CCP4 program suite. A total of 18 Se atoms were found with the autobuild software from Phenix package (Adams et al., 2010). The automatic and manual refinement were performed using REFMAC5 and Coot softwares, respectively (Murshudov et al., 1997; Emsley and Cowtan, 2004). Finally, the *R*_free_ and *R*_work_ were refined to 0.1894 and 0.1471, respectively. The final model was checked by Procheck software and was deposited in the Protein Data Bank with ID number: 5Z3K. The refine statistics were summarized in Table 1. The structural models of CmGH1 complex with substrates and products were built using AutoDock Tools4 program^1^ (Morris et al., 2009).

## Data Availability Statement

The datasets generated for this study can be found in the structure of CmGH1 was deposited in Protein data bank (PDB, http://www.rcsb.org/). The PDB id is 5Z3K.

## Author Contributions

YS, ZL, Y-YH, LB, PX, BG, XH, and X-WX performed the experiments and analyzed the data. JL designed the study and wrote the manuscript.

## Conflict of Interest

The authors declare that the research was conducted in the absence of any commercial or financial relationships that could be construed as a potential conflict of interest.

## Footnotes

1 http://autodock.scripps.edu/
