# Supplementary material for: Structural and Functional Insights Into CmGH1, a Novel GH39 Family β-Glucosidase From Deep-Sea Bacterium
Source: Front Microbiol. 2019 Dec 20;10:2922. doi: 10.3389/fmicb.2019.02922 (PMC6933502; doi:10.3389/fmicb.2019.02922)
Supplement: Supplementary file 1 [file Table_1.DOCX]

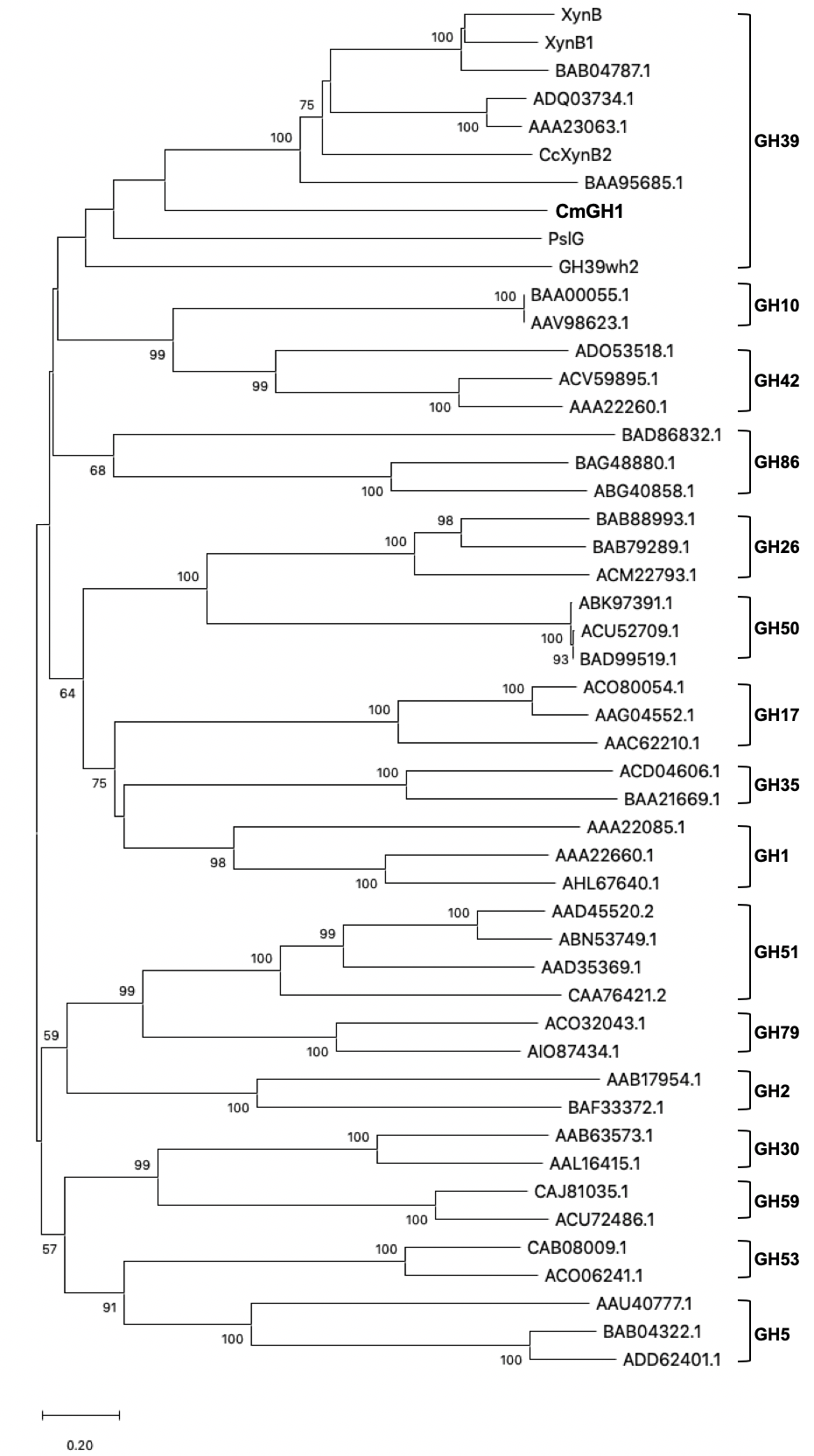


**Figure S1 Neighbor-joining phylogenetic tree of CmGH1.** The tree was constructed using MEGA software. Bootstrap values were based on 1000 replicates and only values >50% were shown. The scale bar indicated the number of amino acid substitution per site.


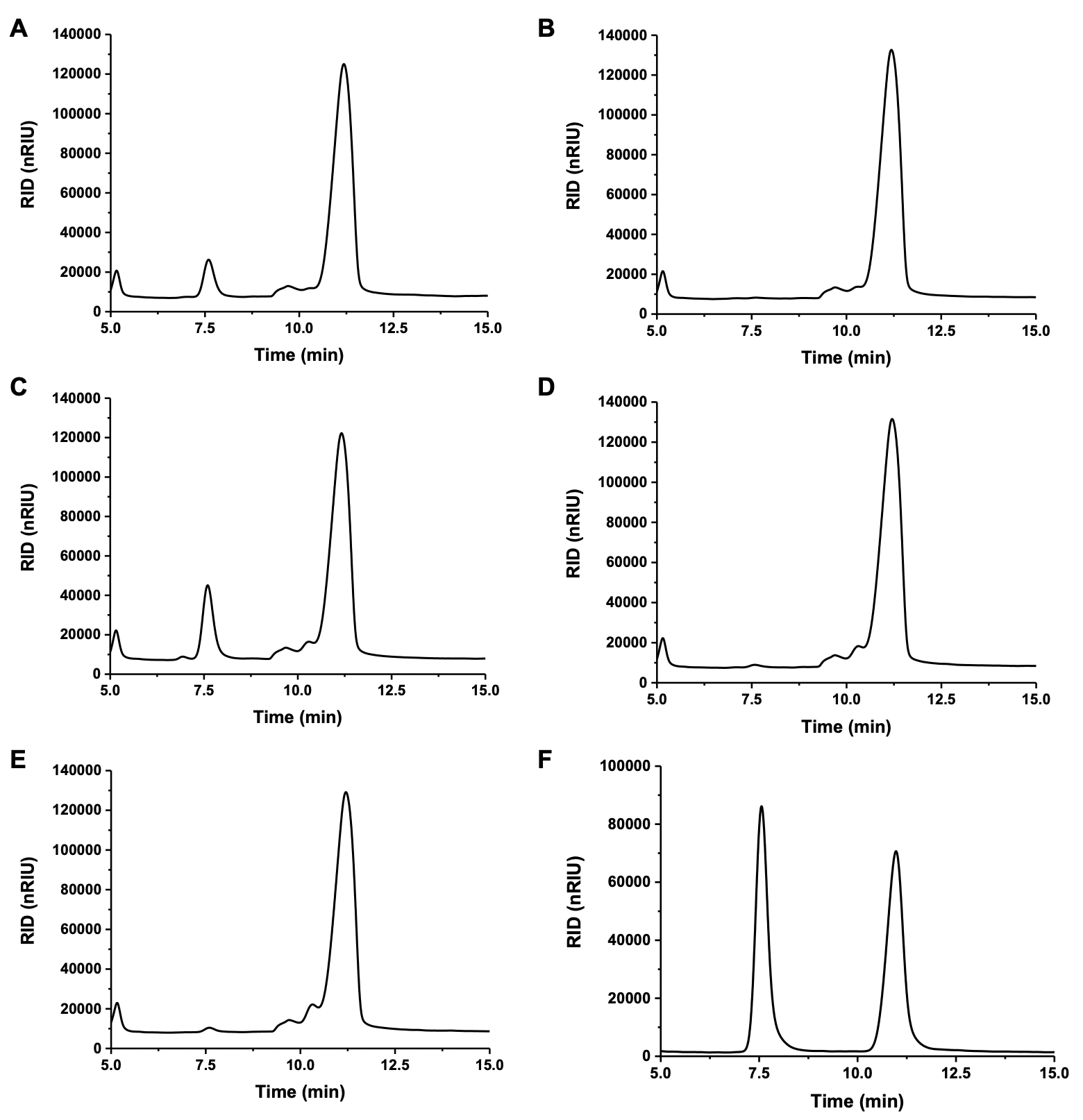


Figure S2 The enzymatic activity of CmGH1 towards cellobiose determined by HPLC. The reaction mixtures at different times were shown in (A) (24 h) and (C) (48 h). Cellobiose and glucose came out at the peak positions of 11.165 min and 7.592 min on a ZORBAX NH_2_ column, respectively. The negative controls with different reaction times were shown in (B) and (D) respectively. Only cellobiose was detected on HPLC. The negative control of reaction mixture, related with Figure 1D, was shown in (E). (F) The standard samples (cellobiose and glucose) were analyzed by HPLC.


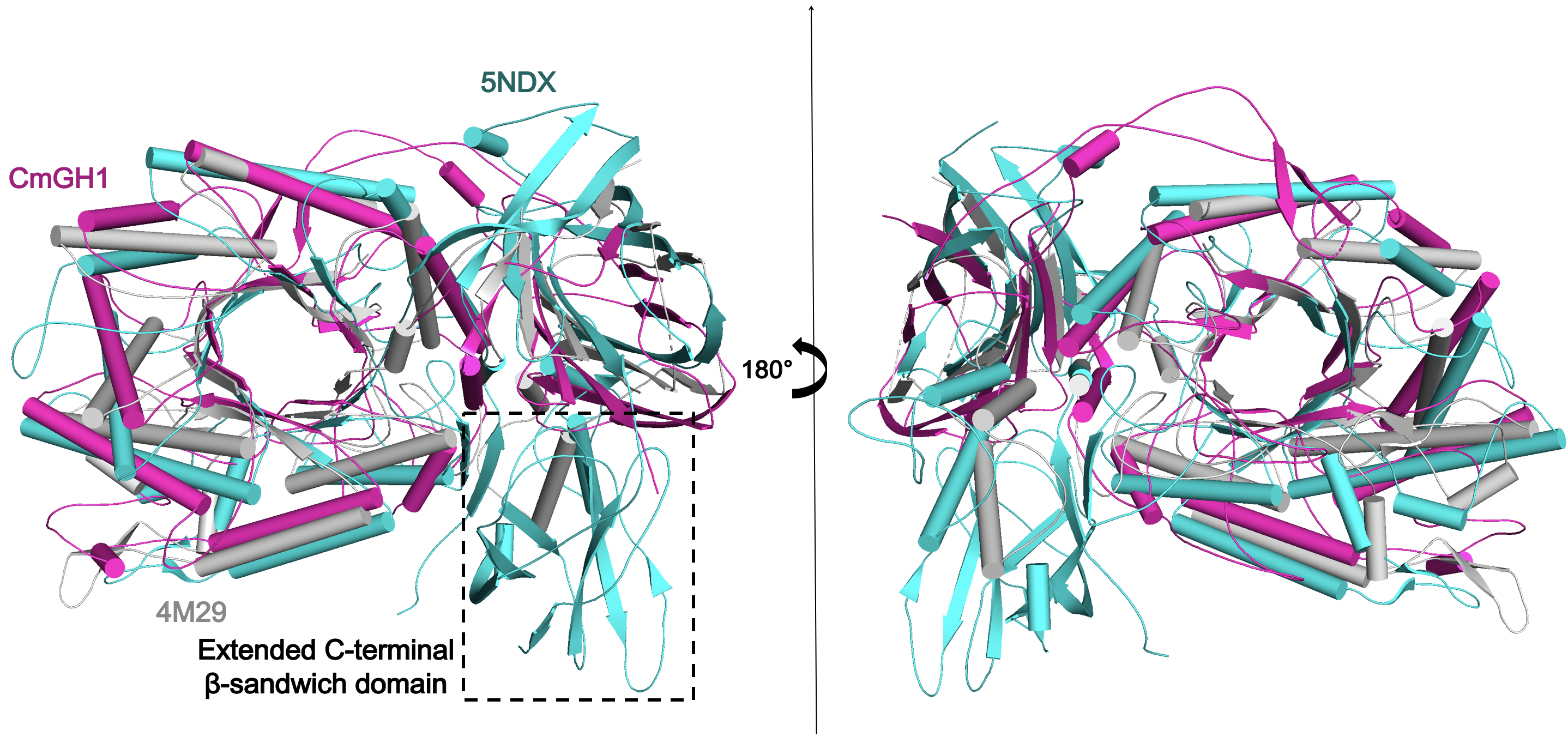


**Figure S3 Structural comparison of CmGH1 with two unpublished homologues of GH39 family.** The two homologues were 5NDX (isolated from *Rhizobium leguminosarum*, RMSD: 12.065 Å, 263 atoms) and 4M29 (isolated from *Caulobacter vibrioides*, RMSD: 7.368 Å, 183 atoms), respectively.


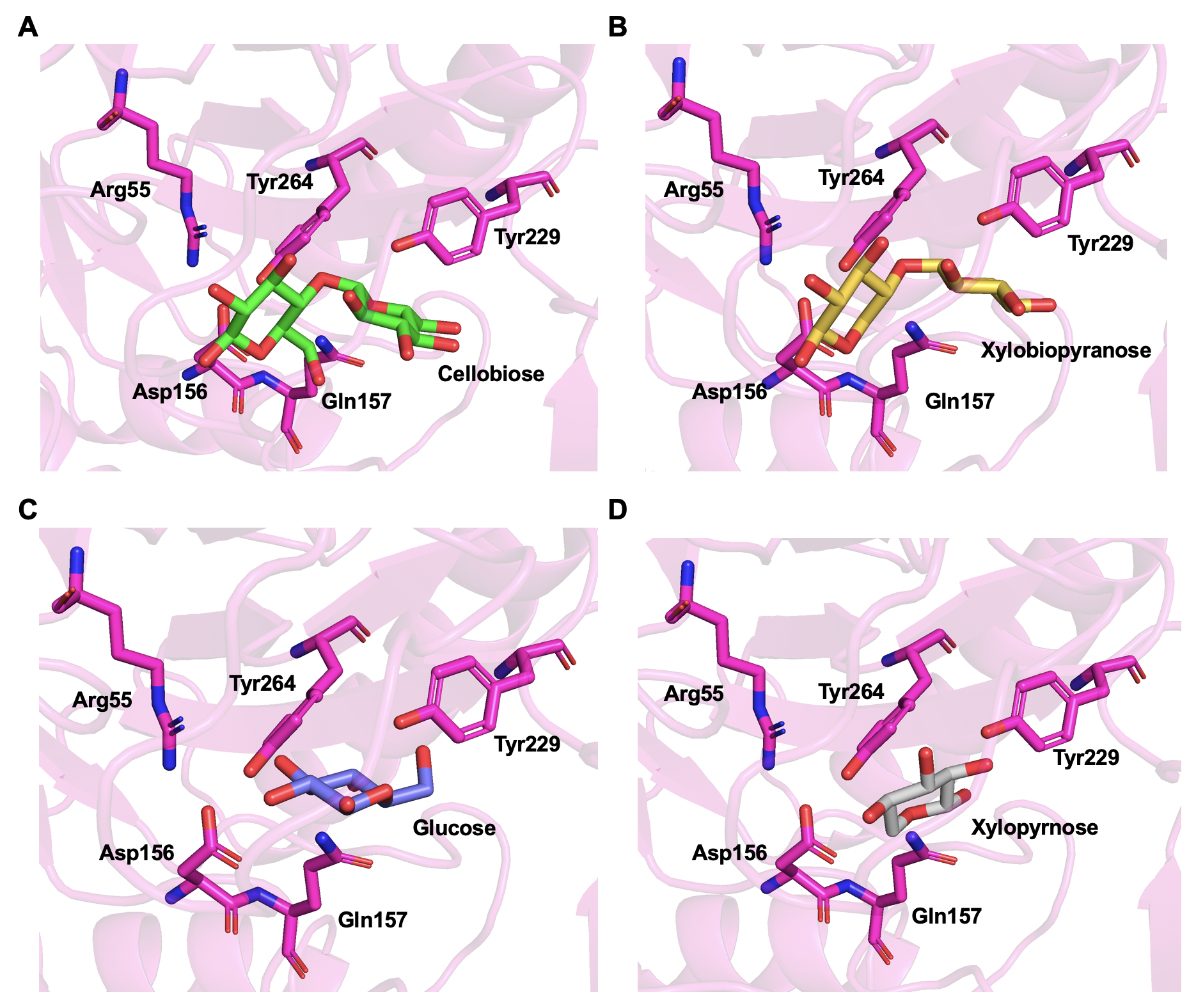


**Figure S4 The models of CmGH1 complexes with different substrates and products.** The structural models of CmGH1 with auto-docked cellobiose, xylobiopyranose, glucose and xylopyranose were shown in A-D, respectively.

**Table S1. The calculated binding energy and inhibition constants of CmGH1 with different ligands**

| ligands | Binding energy (kcal/mol) | KI (mM) |
| --- | --- | --- |
| cellobiose | -4.85 | 0.28 |
| xylobiopyranose | -3.95 | 1.28 |
| glucose | -4.26 | 0.77 |
| xylopyranose | -3.79 | 1.67 |
